# Simulating Immune Interference on the Effect of a Bivalent Glycoconjugate Vaccine against* Haemophilus influenzae* Serotypes “a” and “b”

**DOI:** 10.1155/2016/5486869

**Published:** 2016-03-02

**Authors:** Angjelina Konini, Mingsong Kang, Seyed M. Moghadas

**Affiliations:** Agent-Based Modelling Laboratory, York University, Toronto, ON, Canada M3J 1P3

## Abstract

*Objective.* We sought to evaluate the immune responses to a bivalent* Haemophilus influenzae* glycoconjugate vaccine against serotypes “a” (Hia) and “b” (Hib) in the presence of the preexisting immunity to Hib.* Methods.* We developed a stochastic simulation model of humoral immune response to investigate the antigenic challenge of a bivalent combined glycoconjugate vaccine and a bivalent unimolecular glycoconjugate vaccine. We compared simulation outcomes in the absence of any preexisting immunity with an already primed immune response having specific memory B cells and/or anti-Hib antibodies.* Results.* The simulation results show that the preexisting immune responses to Hib or carrier protein (CP) may significantly impede the production of anti-Hia antibodies by a unimolecular vaccine. In contrast, the production of anti-Hia antibodies using a combined vaccine is inhibited only in the presence of CP immune responses.* Conclusions.* Preexisting immunity to Hib and CP may play a critical role in the development of immune responses against Hia or Hib using bivalent combined and unimolecular vaccine formulations. Our results suggest that a bivalent combined glycoconjugate vaccine with a carrier protein not previously used in Hib conjugate vaccines may be an effective formulation for generating immune responses to protect against both Hib and Hia infections.

## 1. Introduction

*Haemophilus influenzae* is a Gram-negative commensal bacterium, which causes invasive diseases with clinical manifestations such as meningitis, epiglottitis, bacteremia, pneumonia, and septic arthritis, through the invasion into bloodstreams under certain circumstances [1, 2].* H. influenzae* is classified as unencapsulated (nontypeable* H. influenzae*) or encapsulated (serotypes a–f), among which serotype b (Hib) was a leading cause of bacterial meningitis in young children worldwide prior to the introduction of routine infant immunization in the late 1980s [3, 4]. Around 15%–30% of survivors of Hib infection still have some sequela, from hearing impairment to severe permanent neurologic diseases [5–7]. Following the introduction of the Hib glycoconjugate vaccine, the annual incidences of invasive Hib disease and carriage (subclinical infection) in children aged <5 years decreased dramatically in countries where vaccination was implemented [8, 9].

During the preceding decade, incidence rates of other serotypes, particularly serotype a (Hia), have been reported to increase worldwide, especially among the indigenous populations in North America [10–14]. For the invasive Hia disease in Canadian North, a case-fatality rate of 5.6% has been reported [15]. This rate was found to be higher (15.4%) among children with Hia meningitis in Brazil [16] and was found to be of 16% among Canadian pediatric cases [11]. However, due to the lack of comprehensive surveillance programs in many countries, the epidemiological data of Hia-associated diseases are neither complete nor accurately recorded, potentially underestimating the impact of Hia infections worldwide [14].

Based on previous experience with immunizations against Hib, the development of a new vaccine candidate for Hia may be a solution to prevent infection and its severe outcomes. Given the chemical similarities between Hia and Hib capsular polysaccharides, it has been suggested that a bivalent Hib-Hia glycoconjugate vaccine formulation with a similar CP previously used for Hib vaccine could be utilized to induce effective immune protection against both Hia and Hib infections [14, 17]. In this context, however, evidence is accumulating that the glycoconjugate vaccines may elicit low titres of protective antibody against bacterial polysaccharides [18–20]. There have been a number of mechanisms proposed to explain the suboptimal immune responses to this type of vaccine, including the “carrier-induced epitopic suppression” (CIES) [18]. In CIES, polysaccharide antigens conjugated with CP will be rapidly depleted by binding to preexisting anti-CP antibodies and forming the immune complexes, which eventually undergo phagocytosis by phagocytes, such as microphages or dendritic cells. Concurrently, CP-specific memory B cells quickly bind and internalize CP-linked polysaccharide antigens [18, 19]. These processes will in turn impede the polysaccharide-naïve B cell stimulations and therefore interfere with survival and proliferations during clonal expansion as a result of rapid depletion of free antigens [18]. Therefore, we hypothesize that the production of Hia-specific antibodies using a bivalent Hia-Hib vaccine is diminished in the presence of preexisting immune responses against CP or Hib.

At present, no bivalent vaccines against* H. influenzae* serotypes “a” and “b” have been developed, and experimental evaluations of pathogen-specific immune responses to analyze immune interference induced by preexisting immunity are infeasible. We therefore developed a stochastic simulation model of humoral immune response to encapsulate the biological processes underlying T cell-dependent B cell activation and the antibody production. Using this model, we sought to evaluate the potential level of immune responses conferred by a bivalent combined (Hib-CP/Hia-CP) glycoconjugate vaccine and a bivalent unimolecular (Hib-CP-Hia) glycoconjugate vaccine [21], in the presence of preexisting immunity to serotype “b” of* H. influenzae* and CP.

## 2. Methods

**Figure 1:**
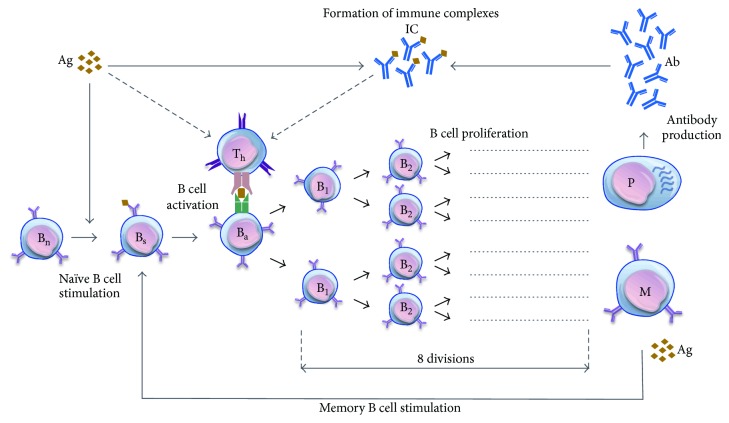
The biological model of humoral immune response. The model includes antigens (Ag), antibodies (Ab); naïve B cells (B_n_), stimulated B cells (B_s_), activated B cells (B_a_), and proliferating B cells (B_1_–B_8_); immune complexes (IC); memory B cells (M); plasma cells (P); and T helper cells (T_h_). Arrows show the transitions between biological states. The dashed-line arrows show multistep processes involved in the biological mechanisms. Naïve B cells are stimulated at the rate *γ*_n_ (through antigen interaction) and activated at the rate *μ*_n_ (through T cell interaction). Proliferation of activated B cells occurs after activation for 8 divisions, leading to the production of plasma and memory B cells. Plasma cells produce antibodies at the rate *η*. Memory cells can be stimulated and activated at the rates *γ*_m_ and *μ*_m_, respectively, in the presence of free antigen peptides and enter the proliferation process. Parameter values for simulating this biological model are presented in Table 1.

To simulate the immune response and antibody production, we developed a stochastic simulation model based on immunological mechanisms of T cell-dependent B cell proliferation. The humoral immune response is initiated upon the recognition of antigens by antigen-presenting cells (APCs), which activate naïve T cells in the form of T helper cells. These helper cells activate stimulated B cells that have already presented the same antigens on the cell surface via major histocompatibility complex class II (MHC II). Activated B cells subsequently proliferate and differentiate into plasma cells (that secrete antibodies) or long-lived memory B cells (for the secondary responses to the same antigenic challenge). In the presence of antigens, memory cells can further be stimulated and enter the cycle of clonal expansion and antibody production. Following secretion, antibodies can bind to antigens to form immune complexes, which can be recognized and cleared by phagocytes (Figure 1).

### 2.1. Stochastic Simulation Model

We implemented a stochastic Markov-Chain simulation model, in which events occur randomly based on the rates of immunological mechanisms. The state of the system is defined by the number of cells, antigens, antibodies, and immune complexes and is changed discretely whenever an event occurs. In our simulations, rates of immunological mechanisms are converted to probabilities of the corresponding event by considering

(1) $$\begin{array}{c} P\left(\;\text{event }i\;\right)=\frac{a_{i}}{\sum_{i}^{}a_{i}}, \end{array}$$

where *a*_*i*_ is the transition rate of the event *i*. In this formulation, the time to the next event (*τ*) is exponentially distributed with the parameter equal to the sum of the rates for all possible events. The probability density function is given by

(2) $$\begin{array}{c} f\left(\;\tau \;\right)=\left(\;\overset{}{\underset{i}{\sum }}a_{i}\;\right)\exp\left(\;-\tau \overset{}{\underset{i}{\sum }}a_{i}\;\right). \end{array}$$

Using inverse transform sampling [22], we estimated the time to the next event. For a given random variate *r* drawn from the uniform distribution on the unit interval (0,1), *τ* was estimated as −ln⁡*r*/∑_*i*_ *a*_*i*_. To determine the nature of the next event, we ordered the events as an increasing fraction of the sum of all events and compared with another uniform deviate generated in the unit interval. Simulations were run for a large number of samples (*n* = 500) to calculate the average of sample realizations of the stochastic process in each scenario.

### 2.2. Parameterization

We parameterized the model using available estimates from the previous literature on the development of humoral immune responses (Table 1). Since the specific interactions between T cells and macrophages have been measured in a short (5 to 15 minutes) time period [23], we used an average value of 10 minutes to calculate the MHC II antigen presentation rate, giving a rate of 6 h^−1^. Naïve B cells are stimulated and activated at a much slower rate compared to memory B cells [24, 25]. We used rates of 5.26 × 10^−2^ and 0.5 h^−1^ for naïve and memory B cells activation, respectively. Each division during proliferation cycle of immune cells takes about 8 hours [26], and we used a rate of 0.125 h^−1^. Upon the completion of each division cycle, plasma B cells are generated, which secrete antibodies at an estimated rate of around 2000 molecules per second [27]. This gives the rate of 7.2 × 10^6^ antibody molecules per cell per hour. The binding rate of antibody-antigen is taken from the previous literature considering the affinity and the number of binding sites of the antibodies [28]. High avidity antibodies will react rapidly, while low avidity species may continue to form complexes for several hours after the initiation of reaction. Since the peak rate of immune complex formation occurs within 1 minute after mixing antibodies and antigens [28], we used an antigen-antibody binding rate of 60 h^−1^ per antigen. We used an average life span of short- and long-lived plasma cells [29–31], with a rate of 8.33 × 10^−3^ h^−1^ within the reported ranges. For the purpose of simulations, an initial number of 4 × 10^4^ antigens was used in four different scenarios: (i) naïve immune system, (ii) preexisting antibodies (Ab = 10^6^ molecules) against only one antigen, (iii) preexisting memory B cell (M = 400 cells) specific to only one antigen, and (iv) preexisting antibodies and memory B cells (Ab = 4 × 10^3^ molecules and M = 200 cells) specific to only one antigen.

## 3. Results

**Figure 2:**
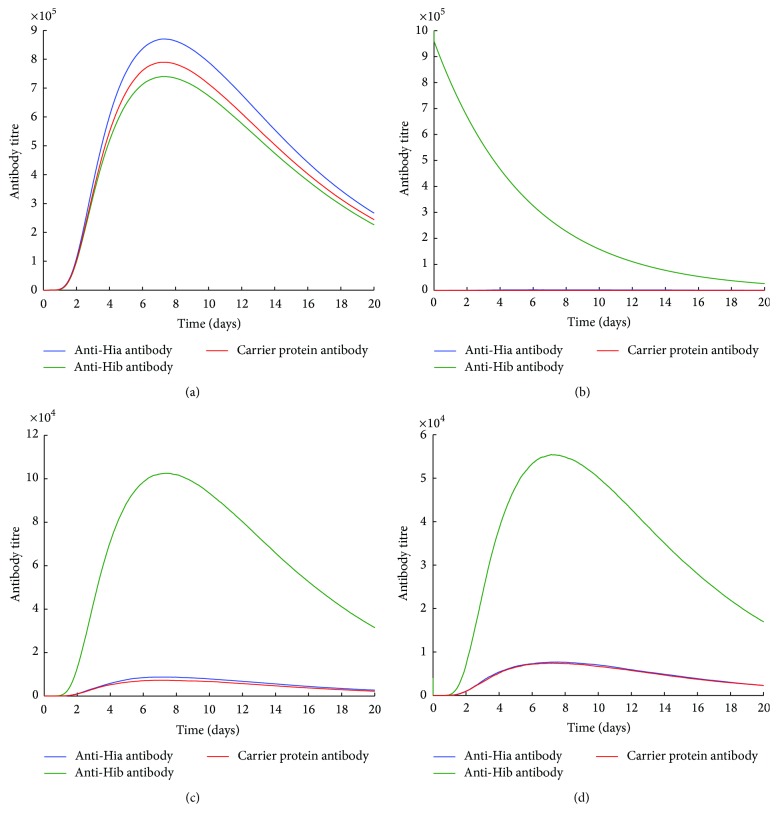
The effects of preexisting Hib immune responses on the production of antibodies using a bivalent unimolecular Hib-CP-Hia vaccine. An initial 4 × 10^4^ amount of antigen was used for simulations (a) in the naïve condition (preexisting Ab = 0 and M = 0), (b) in the presence of preexisting anti-Hib antibodies only (preexisting Ab^b^ = 10^6^), (c) in the presence of preexisting Hib-specific memory B cells only (M_b_ = 400), and (d) in the presence of both anti-Hib antibodies and Hib-specific memory B cells (Ab = 4 × 10^4^ and M_b_ = 200).

**Figure 3 fig3:**
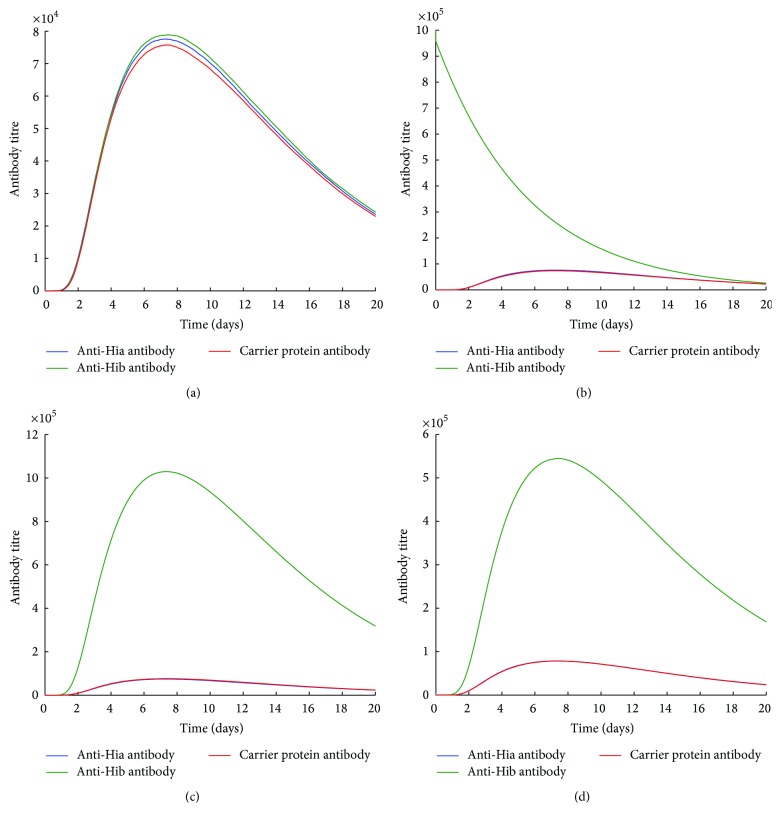
The effects of preexisting Hib immune responses on the production of antibodies using a bivalent combined Hib-CP/Hia-CP vaccine. An initial 4 × 10^4^ amount of antigen was used for simulations (a) in the naïve condition (preexisting Ab = 0 and M = 0), (b) in the presence of preexisting anti-Hib antibodies only (preexisting Ab^b^ = 10^6^), (c) in the presence of preexisting Hib-specific memory B cells only (M_b_ = 400), and (d) in the presence of both anti-Hib antibodies and Hib-specific memory B cells (Ab = 4 × 10^4^ and M_b_ = 200).

**Figure 4:**
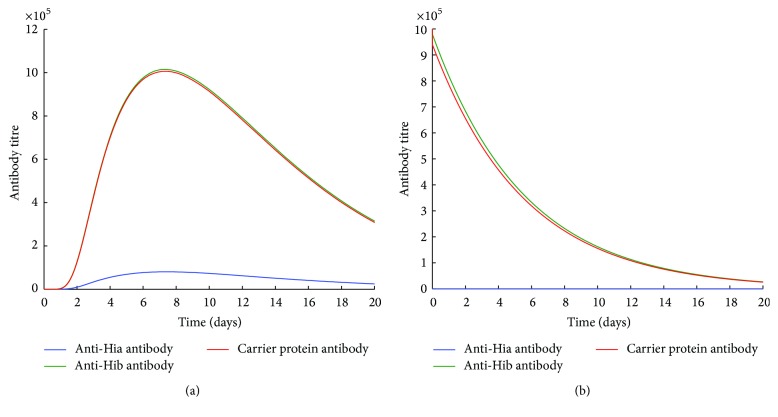
The effects of preexisting CP and Hib immune responses on the production of antibodies using a bivalent combined Hib-CP/Hia-CP vaccine. An initial 4 × 10^4^ amount of antigen was used for simulations (a) in the presence of preexisting CP-specific and Hib-specific memory B cells only (M_b_ = 400 and M_CP_ = 400) and (b) in the presence of preexisting anti-Hib and CP antibodies only (Ab = 10^6^ and Ab_CP_ = 10^6^).

Simulation results for antibody titres using the bivalent combined and unimolecular glycoconjugate vaccines are illustrated in Figures 2 [Fig fig3]–4. In a naïve condition (Figures 2(a) and 3(a)), both Hib-CP-Hia and Hib-CP/Hia-CP can elicit comparable levels of anti-Hia, anti-Hib, and CP-specific antibodies. In the presence of preexisting Hib-specific immune responses, such as antibodies and/or memory B cells, the production of anti-Hia and CP antibodies by a unimolecular Hib-CP-Hia vaccine is significantly reduced (Figures 2(b)–2(d)), compared to the scenario of the naïve condition. Furthermore, preexisting Hib antibodies may impede the boosting of Hib-specific antibodies following vaccination with both Hib-CP-Hia and Hib-CP/Hia-CP formulations (Figures 2(b) and 3(b)).

In contrast, the preexisting Hib-specific immune responses have no effect on the production of anti-Hia antibodies by a combined Hib-CP/Hia-CP vaccine (Figures 3(b)–3(d)). However, when immune responses of CP and Hib-specific antibodies or memory cells exist, the generation of Hia-specific antibodies is largely inhibited (Figures 4(a) and 4(b)). These simulations suggest that the preexisting CP-specific immune responses may significantly impede the production of both anti-Hia and anti-Hib antibodies when a bivalent vaccine contains the same CP. However, the preexisting Hib-specific antibodies or memory cells may interfere with and impede the development of anti-Hia immune responses only in a bivalent unimolecular vaccine.

## 4. Discussion

Recent studies indicate differential rates of Hia incidence in terms of morbidity and severe outcomes in several population settings, including aboriginal people in North America [11, 14, 32–35]. Hia prevention in these populations has therefore become an important public health concern. Given the potential for Hia spread in other population settings, the development of a bivalent Hia-Hib conjugate vaccine has been proposed for the prevention of both Hia and Hib infections [14, 17].

In this study, using a simulation model, we evaluated the potential effect of immunization with such a bivalent vaccine on the elicitation of immune responses. Our simulations suggest that, in either type of bivalent combined or unimolecular vaccine, the preexisting immunity to one antigen at the individual level may interfere with the production of antibodies against both antigens. In particular, the preexisting CP or Hib-specific antibodies or memory B cells respond faster to the same antigen (CP or Hib) than naïve B cells, which leads to rapid depletion of free antigens through the interaction with memory B cells or the formation of immune complexes. This will in turn impede the generation of anti-Hia antibody production (Figures 2(c), 2(d), 3(a), 3(b), 4(a), and 4(b)) or even reduce the titre of preexisting Hib antibodies (Figures 2(b), 3(b), and 4(b)). However, in a naïve individual with no prior exposure or vaccination, a bivalent vaccine can trigger comparable immune responses against both Hia and Hib (Figures 2(a) and 3(a)).

These findings have important implications for current efforts towards vaccine development against Hia, as well as vaccination policies. Given the fact that Hib and Hia have been circulating in several regions of the world [14, 36] and the Hib conjugate vaccine has been included in the universal infant immunization programs in a number of countries since the late 1980s [36], our results indicate that the use of a bivalent Hia-Hib vaccine in these population settings (where individuals may have varying degrees of preexisting immunity due to natural infection or vaccination) may not be effective in raising antibody titres to levels required for Hia prevention. While such a vaccine may be recommended for immunization of infants (as naïve individuals) to prevent the spread of both Hia and Hib, the duration of vaccine-induced protection remains undetermined, and therefore booster doses may be required [37]. Our recent study also demonstrates that when a monovalent Hia vaccine becomes available, achieving and maintaining a sufficiently high level of herd immunity for curtailing Hia requires vaccination of a fraction of susceptible individuals in addition to high primary and booster vaccine coverage of newborns [38].

Given the previous clinical and laboratory investigations of CIES, a new bivalent vaccine formulation may be optimized to enhance Hia-specific immune responses and antibody production* via* various approaches, such as the utilization of a different carrier protein from the one previously used for the Hib conjugate vaccine or the inclusion of high-density Hia polysaccharide antigens [19, 39]. The use of a different CP from the one previously used can eliminate the potential effects of preexisting CP immune responses on the production of anti-Hia antibodies elicited by a bivalent combined vaccine (Hia-CP/Hib-CP). However, in a bivalent unimolecular vaccine (Hia-CP-Hib), a high density of Hia antigens may increase the probability of recognition by Hia-specific naïve B cells. Our findings in this study (and additional simulations) suggest that, for the development of a bivalent combined glycoconjugate vaccine, the utilization of a different carrier protein from the one previously used for Hib vaccine can help develop effective vaccine-induced protections against both Hib and Hia infections.

Our study has several limitations that warrant further investigation. For simulations presented here, we have relied on parameter estimates reported in the previous literature, which may be subject to variations, and we therefore emphasize the qualitative aspects of this study. Our model is also subject to some limitations due to simplifying assumptions on the mechanisms involved in the development of humoral immune responses. The preexisting immunity in our model was included as the initial condition for antibodies or memory B cells at the time of vaccination without considering any prior specific mechanisms for their generation. However, we note that antigens from several organisms other than Hia and Hib can induce cross-reactive antibodies to* H. influenzae* capsular polysaccharide [40]. Despite these limitations, our study provides the first simulation model for evaluation of the bivalent vaccine-induced immunity, which can be extended with further immunological mechanisms and recalibrated as data become available through further immunological experiments.

## 5. Conclusions

Our results highlight the importance of preexisting immune responses to Hib and CP (conferred by natural infection or vaccination) for the composition of a new bivalent vaccine against Hia and Hib. The findings indicate that the use of new carrier proteins that are not previously included in the Hib conjugate vaccine may be a viable approach for the development of an effective bivalent combined vaccine. In this context, ongoing efforts should include experimental investigation of the immune responses elicited by bivalent vaccines in the presence of preexisting immunity.

## Figures and Tables

**Table 1:** Description of model parameters and their values.

Parameter	Description	Value	References
*γ*_n_	Stimulation rate of naïve B cells	0.5 (antigen)^ −1^ h^−1^	[24]
*μ*_n_	Activation rate of B cells	5.88 × 10^−2^ (MHC II)^−1^ h^−1^	[25]
*γ*_m_	Stimulation rate of memory B cells	1 (antigen)^ −1^ h^−1^	Estimated
*μ*_m_	Activation rate of memory B cells	1 (MHC II)^−1^ h^−1^	Estimated
*α*	Rate of MHC II antigen presentation	6 h^−1^	[23]
*β*	Binding rate of antibody with antigen	60 (antigen molecule)^ −1^ h^−1^	[28]
*δ*_n_	Death rate of naïve B cells	4.16 × 10^−3^ h^−1^	[31]
*δ*_m_	Death rate of memory B cells	2.1 × 10^−4^ h^−1^	Estimated
*δ*_a_	Activation-induced cell death rate in the absence of antigen	6.67 × 10^−2^ h^−1^	[31]
δ_p_	Death rate of plasma B cells	8.33 × 10^−3^ h^−1^	[29–31]
*δ*_b_	Antibody removal rate	8.33 × 10^−3^ h^−1^	[31]
*d*_g_	Antigen removal rate	8.33 × 10^−3^ h^−1^	Estimated
*ρ*	Production rate of naïve B cells from bone marrow	10 day^−1^	[41]
*η*	Rate of antibody production from plasma B cells	7.2 × 10^6^ (cell)^−1^ h^−1^	[27]
